# Successful Resection of Gastric Subepithelial Lipoma Using the Bite-on-Bite Approach: Reviving Old Techniques in a Peripheral Hospital

**DOI:** 10.7759/cureus.40151

**Published:** 2023-06-08

**Authors:** Serbulent Aydin, Mohamed Elgamal, Yucel Aydin

**Affiliations:** 1 General Surgery, Bursa Orhaneli State Hospital, Bursa, TUR; 2 Internal Medicine, St. Mary's Hospital, Waterbury, USA; 3 Department of Medicine, St. Mary's Hospital, Waterbury, USA

**Keywords:** endoscopic mucosal resection, dyspepsia, endoscopy, lipoma, submucosal lesions

## Abstract

Subepithelial lesions (SELs) are common findings in the gastrointestinal (GI) tract. They are often benign and asymptomatic but can cause symptoms in some cases. The approach to endoscopic management of these lesions depends on various factors, including associated symptoms, location, available equipment, and operator expertise. In this case report, we present a 50-year-old male with long-standing dyspepsia who was found to have a submucosal lesion in the stomach. The lesion was successfully treated using the bite-on-bite method with cold biopsy forceps. This report aims to discuss gastric subepithelial lesions and current management options, and highlight an old technique for endoscopists in the era of advanced endoscopy.

## Introduction

Subepithelial lesions (SELs) are tumors that originate from the layers beneath the superficial mucosa in the gastrointestinal (GI) tract, including the muscularis mucosa, submucosa, or muscularis propria. Localization, mucosal characteristics, and evaluation using advanced endoscopy techniques such as endoscopic ultrasonography (EUS) along with tissue acquisition via fine needle aspiration (FNA) or biopsy (FNB) are crucial for identifying and definitively diagnosing subepithelial masses in the GI tract [1].

The stomach is the most common site for SELs, particularly lipomas, leiomyomas, gastrointestinal stromal tumors (GISTs), and pancreatic rests in the upper GI tract. The incidence and prevalence of SELs in the upper GI tract can vary depending on the population and data sources. Approximately 1% of all upper GI endoscopic examinations diagnose a subepithelial lesion. GISTs have an estimated incidence of 10 to 20 cases per million individuals per year. However, the overall prevalence of GISTs in the general population is relatively low (around 10 to 20 cases per 100,000 individuals). GI tract leiomyomas are more common than GISTs, but determining their exact incidence and prevalence is challenging due to their often asymptomatic nature. Lipomas are frequently detected incidentally during diagnostic procedures in the upper GI tract and are relatively prevalent. Pancreatic rests in the upper GI tract are present in approximately 1.2% of the general population [2]. However, most SELs are considered asymptomatic, as they are often incidentally discovered during upper GI endoscopy performed for other reasons. Symptomatic SELs present with overt or occult GI bleeding, iron deficiency anemia, refractory dyspepsia despite proton pump inhibitor use, or obstruction symptoms such as nausea, vomiting, and weight loss require endoscopic evaluation [3].

Gastric lipomas account for 2-3% of all benign gastric tumors [4,5]. During the initial procedure, the consistency of an SEL should be assessed using closed biopsy forceps to determine the presence of the "pillow" or "cushion" sign, which is highly specific (98%) for diagnosing lipomas [6]. Surgical approaches or advanced endoscopy techniques such as endoscopic submucosal dissection are often used for deep tissue biopsy or removal of SELs [7]. The bite-on-bite method, a rarely reported technique for SEL removal, was employed successfully in this case. We present the case of a 50-year-old gentleman with refractory dyspepsia who underwent upper GI endoscopy and was found to have an antral SEL consistent with a lipoma. The lipoma was resected successfully using the bite-on-bite technique without complications at a peripheral hospital.

## Case presentation

A 50-year-old male presented to the general surgery department with complaints of epigastric discomfort, early satiety, and nausea persisting for 6 months, despite taking pantoprazole 40 mg twice daily for the past 4 weeks. His medical history included hyperlipidemia and hypertension, for which he was taking atorvastatin 20 mg and ramipril 5 mg daily, in addition to pantoprazole. Upon examination, his vital signs, physical examination, and laboratory data were unremarkable.

**Figure 1:**
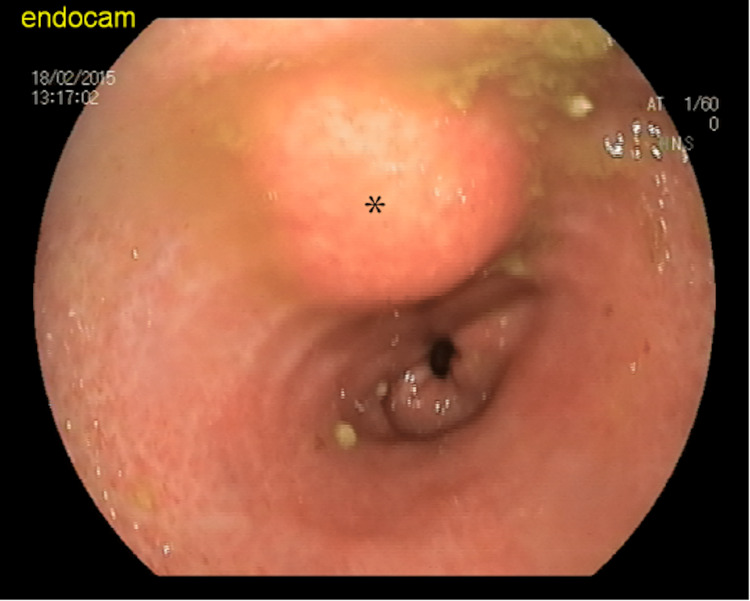
Normal endoscopic mucosa and SEL Asterisk (*) indicates SEL.

An upper GI endoscopy revealed a 3x3 cm SEL with normal overlying mucosa (Figure 1).

**Figure 2:**
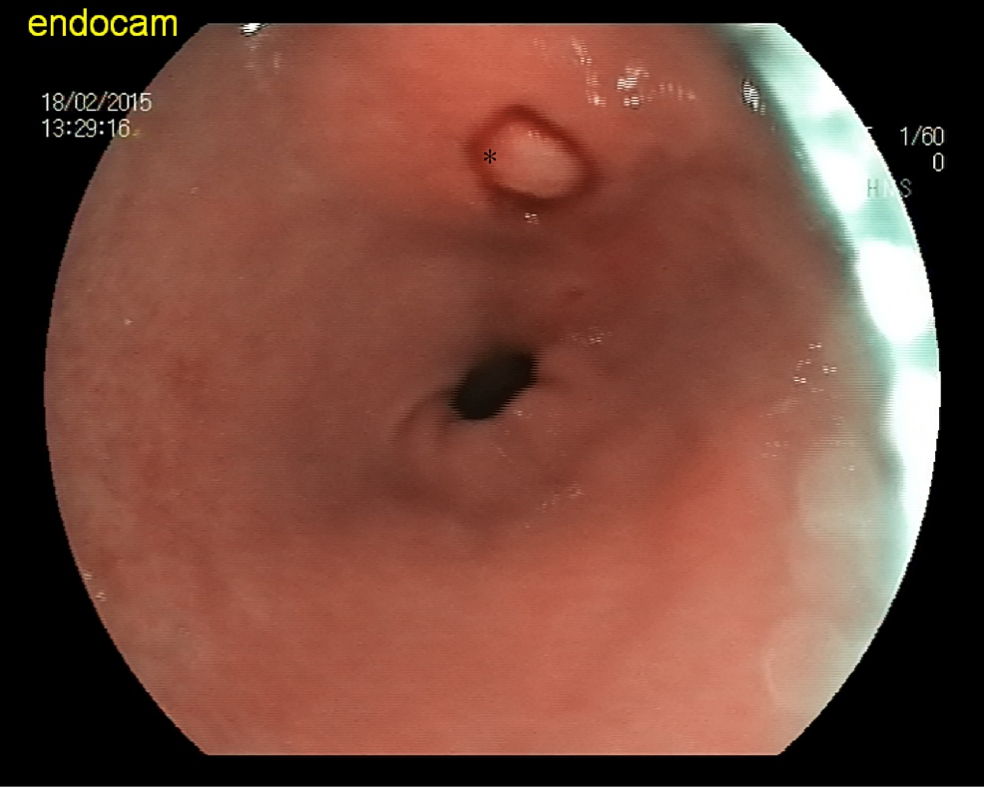
Asterisk (*) demonstrates yellowish sparkling fat and a capsule of SEL. A Repeat bite-on-bite method was carried out to expose SEL.

Initially, an attempted biopsy of the covering normal mucosa using cold biopsy forceps exposed an underlying mass consistent with a lipoma. The lipoma exhibited yellowish sparkling fat and a capsule. Subsequently, the bite-on-bite method was performed to uncover the upper part of the mass (Figure 2).

**Figure 3:**
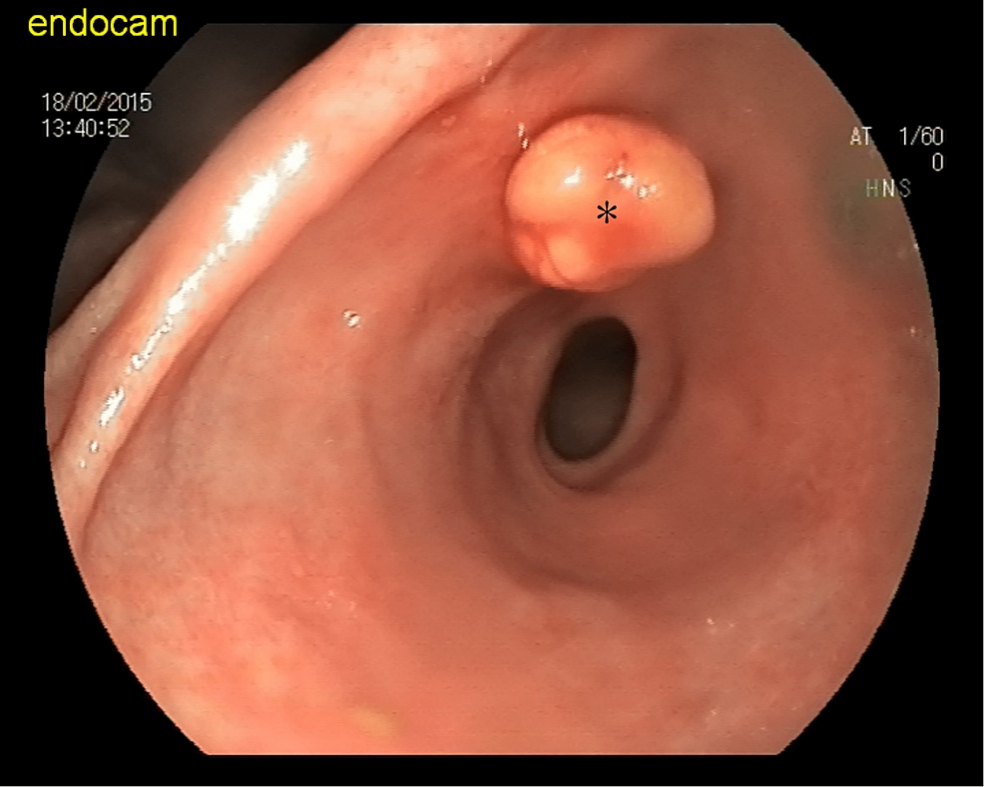
Asterisk (*) shows the lipoma that was successfully removed from the gastric wall with cold biopsy forceps.

The lipoma was successfully removed by pulling it up and down using cold biopsy forceps (Figure 3).

**Figure 4:**
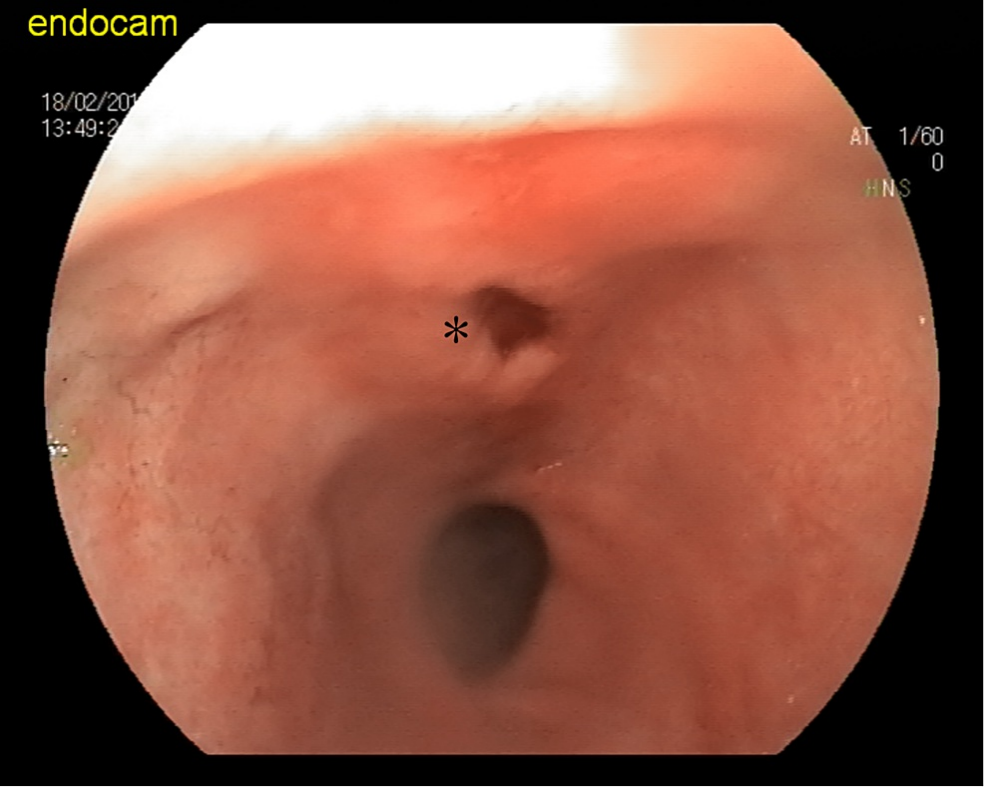
Post-procedure area is marked with an asterisk (*). Lipoma removal by cold biopsy forceps did not cause any complications including bleeding as shown.

No complications such as bleeding or perforation were observed following the avulsion of the lesion from the gastric wall (Figure 4).

**Figure 5:**
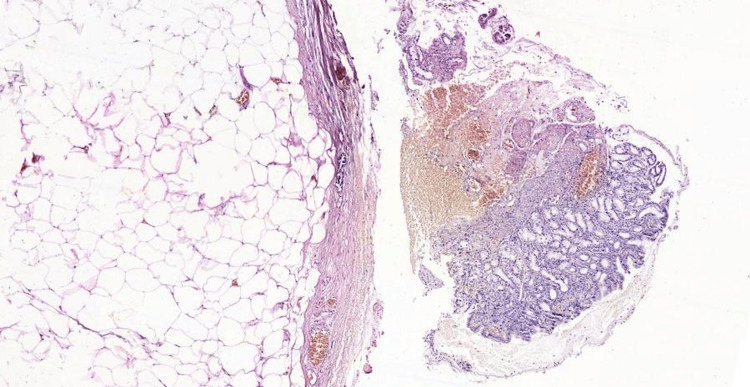
Hematoxylin and eosin staining of the lesion with capsule and mature adipocytes. The overlying gastric mucosa is normal. Magnification 10X

Histopathology confirmed the diagnosis of a lipoma (Figure 5). The histopathologic examination of randomly selected stomach biopsies did not demonstrate the presence of *Helicobacter pylori*. Considering the consistent findings of SEL resembling a lipoma and the highly specific endoscopic appearance supporting the diagnosis, the decision to forgo computerized tomography (CT) imaging was justified.

## Discussion

SELs in the GI tract are commonly encountered during routine endoscopic examinations. These lesions are often incidental findings due to their asymptomatic and benign nature. However, when the lesion size is equal to or greater than 20 mm, further evaluation for tissue diagnosis is warranted. Various techniques, such as EUS-guided fine needle biopsy or mucosal incision-assisted biopsy (MIAB), have been employed to obtain adequate tissue samples for histological examination [5]. In addition to obtaining tissue samples, EUS offers the advantage of assessing the vascularity (blood flow) of the suspicious lesion. Doppler ultrasound is commonly employed during EUS to evaluate the vascularity of the lesion. This information can be instrumental in determining the nature of the lesion, enabling differentiation between benign and malignant masses [8].

MIAB has emerged as a reliable and effective approach for the diagnosis of SELs. It is particularly suitable for lesions smaller than 20 mm where the overlying mucosa is expected to be histologically normal. The bite-on-bite technique is one such MIAB approach that allows for high-quality tumor tissue acquisition for pathologic evaluation [9]. This technique involves creating an incision in the mucosa overlying the lesion and using a specialized device to bite into the lesion, ensuring sufficient tissue capture for accurate diagnosis [10].

Regular biopsy forceps allow for obtaining smaller tissue samples but have limited grasping capacity and carry a risk of tissue trauma. On the other hand, jumbo forceps offer increased grasping capacity and enhanced stability but are less maneuverable and may reduce dexterity. The choice of forceps depends on procedural requirements and anatomical factors. For this particular procedure, regular biopsy forceps were selected due to their superior handling precision and maneuverability. We demonstrated the successful use of the bite-on-bite technique for the safe removal of a subepithelial lesion, obviating the need for a separate biopsy procedure. This approach offers several advantages, especially in settings where advanced endoscopic equipment may not be readily available, such as peripheral hospitals. By utilizing the bite-on-bite technique, endoscopists can effectively obtain tissue samples for diagnosis while minimizing the need for additional invasive procedures.

The bite-on-bite technique has shown promising results not only for SELs but also for other submucosal lesions, including pancreatic rests and neuroendocrine tumors, in symptomatic cases. By incorporating this technique into their practice, endoscopists can potentially improve diagnostic accuracy and reduce the need for more invasive interventions. However, it is essential to exercise caution and adhere to proper patient selection criteria, lesion characteristics, and endoscopic expertise to minimize the risk of complications. While the reported incidence of major complications, such as bleeding or perforation, associated with the bite-on-bite technique is low, it is crucial to remain vigilant and adhere to strict procedural guidelines [11].

## Conclusions

In summary, gastric SELs pose a diagnostic challenge due to their diverse nature and potential for malignant transformation. While advanced endoscopic techniques such as EUS and endoscopic submucosal dissection have revolutionized the field, their availability remains limited in certain healthcare settings. According to the current guidelines provided by the American College of Gastroenterology (ACG) for the management of gastric SELs, the statements regarding the use of the bite-on-bite technique using cold biopsy forceps are not aligned with the recommended approach. The ACG guidelines do not recommend the utilization of bite-on-bite biopsies for the evaluation of SEL prior to the EUS examination. Instead, the guidelines emphasize the importance of conducting a EUS evaluation for SEL in order to assess the extension of the lesion, identify any vascular involvement, and evaluate the surrounding anatomical structures. However, careful patient selection, appropriate lesion characterization, and adherence to procedural guidelines are essential to ensure optimal outcomes.

## References

[1] Siddiqui A, Kunda R, Tyberg A (2019). Three-way comparative study of endoscopic ultrasound-guided transmural gallbladder drainage using lumen-apposing metal stents versus endoscopic transpapillary drainage versus percutaneous cholecystostomy for gallbladder drainage in high-risk surgical patients with acute cholecystitis: clinical outcomes and success in an International, Multicenter Study. Surg Endosc.

[2] Hedenbro JL, Ekelund M, Wetterberg P (1991). Endoscopic diagnosis of submucosal gastric lesions. The results after routine endoscopy. Surg Endosc.

[3] Nikolić M, Boban M, Ljubicić N, Duvnjak M, Hrabar D, Pavić T (2009). Evaluation of upper gastrointestinal submucosal lesions by endoscopic ultrasonography. [Article in Croatian]. Acta Med Croatica.

[4] Fernandez MJ, Davis RP, Nora PF (1983). Gastrointestinal lipomas. Arch Surg.

[5] Park SH, Han JK, Kim TK (1999). Unusual gastric tumors: radiologic-pathologic correlation. Radiographics.

[6] Menon L, Buscaglia JM (2014). Endoscopic approach to subepithelial lesions. Therap Adv Gastroenterol.

[7] Lee JS, Kim GH, Park DY (2015). Endoscopic submucosal dissection for gastric subepithelial tumors: a single-center experience. Gastroenterol Res Pract.

[8] Jacobson BC, Bhatt A, Greer KB, Lee LS, Park WG, Sauer BG, Shami VM (2023). ACG Clinical Guideline: diagnosis and management of gastrointestinal subepithelial lesions. Am J Gastroenterol.

[9] Moon JS (2012). Endoscopic ultrasound-guided fine needle aspiration in submucosal lesion. Clin Endosc.

[10] Ehrlich D, Mukewar S, Wang H, Muthusamy VR (2019). Bite-on-bite technique for removal of a gastric subepithelial lipoma. VideoGIE.

[11] Tan Y, Tang X, Huang J, Li R (2022). Efficacy, feasibility, and safety of endoscopic ultrasound-guided fine-needle biopsy for the diagnosis of gastrointestinal subepithelial lesions: a systematic review and meta-analysis. J Clin Gastroenterol.

